# Suicidal Distress and Daily Well-Being: A New Model of Social Hysteresis

**DOI:** 10.3390/bs16020215

**Published:** 2026-02-03

**Authors:** Enrique Fernández-Vilas, Juan José Labora González, Juan R. Coca

**Affiliations:** 1 Department of Sociology and Social Work, University of Valladolid, 42004 Soria, Spain; juanr.coca@uva.es; 2 Department of Political Science and Sociology, University of Santiago de Compostela, 15782 Santiago de Compostela, Spain; juan.labora@usc.es

**Keywords:** daily well-being, social hysteresis, habitus–field mismatch, suicidal distress, anomie, social acceleration

## Abstract

Social acceleration and recurrent structural shocks increase habitus–field mismatch, yet similar exposure does not produce uniform trajectories of daily well-being or suicidal distress. This paper asks how comparable structural strain can generate divergent, path-dependent outcomes and why suicidal vulnerability may persist after objective conditions improve. We develop a theory-building, concept-driven framework that integrates Bourdieu’s practice theory with social and behavioural scholarship on stress, anomie, and despair, and conceptualises these dynamics as social hysteresis. The regime-based model specifies two ideal-typical response orientations through which mismatch can stabilise: an anomic regime marked by shame, withdrawal, and inwardly directed harm, and a radicalising regime marked by grievance framing, moral indignation, and organised participation, without implying violent extremism. Represented through hysteresis loops, the framework implies multistability, asymmetric switching thresholds, and scarring, providing a mechanism for persistence and non-linearity in distress trajectories. The model derives testable expectations for longitudinal panel and experience-sampling designs and suggests that prevention and intervention design should combine reductions in mismatch with relational and institutional infrastructures that facilitate regime shifts and reopen the space of possibles.

## 1. Introduction and Background

Hysteresis is central to this paper’s argument because it captures a specific class of dynamics in which present states depend not only on current conditions but also on prior trajectories. In the social and behavioural sciences, social hysteresis is typically used to describe path-dependent (De Arruda et al., 2023; Sznajd-Weron et al., 2024), non-linear responses in which comparable contemporaneous strain can be associated with different states because the system retains memory of prior exposure and adaptation, yielding multistability and asymmetric switching thresholds. This understanding is increasingly formalised in recent agent-based and opinion-dynamics work (Doniec et al., 2022), where hysteresis appears as a history-dependent mapping between a control variable (e.g., external pressure or shock) and a response variable (e.g., expressed state or behavioural orientation), such that recovery does not simply retrace the path of deterioration (Doniec et al., 2022; Kamińska & Sznajd-Weron, 2025; Sznajd-Weron et al., 2024).

Two properties are especially relevant for behavioural outcomes. First, hysteretic systems often exhibit thresholds and metastable states that permit discontinuous transitions between response orientations (De Arruda et al., 2023; Malizia et al., 2025). Second, they show partial irreversibility (“scarring”) (Gou et al., 2025; Sznajd-Weron et al., 2024): improvements in objective conditions do not necessarily restore prior levels of functioning if the history of exposure sustains residence on a given branch. In this sense, hysteresis is not reducible to a simple temporal lag; it denotes a structured form of non-linearity with multiple stable states, threshold structure, and history dependence.

In sociology, the paradigmatic translation of hysteresis is found in Pierre Bourdieu. The hysteresis effect names the mismatch that arises when habitus, formed under particular historical conditions, confronts a field that has undergone rapid change. Dispositions calibrated to a prior social world become out of phase with the opportunities, demands, and evaluative rules of a transformed environment (Bourdieu, 1977, 1990; Enderle, 2024). These dispositions do not automatically reconfigure in step with structural transformation. They persist and are enacted in contexts where they no longer secure equivalent symbolic or material returns, generating sanctions, misrecognitions, and sustained experiences of practical misfit.

On this reading, hysteresis functions as an analytic concept for theorising temporal disjunction between objective structures and embodied structures. Contemporary distress can be approached as systemic tension: practised schemes continue to respond to social configurations that have lost their former validity. A hysteretic perspective therefore incorporates structural memory into accounts of suffering and response. Biographical trajectories, cumulative crises, and social scarring condition which coping repertoires remain available when new episodes of habitus–field mismatch occur. This is consistent with recent formal work on social hysteresis showing that the sequence of shocks and adaptations can shape present response and delay recovery even when external conditions improve (Sznajd-Weron et al., 2024).

This paper develops the argument in two moves. First, it advances a framework of social hysteresis to account for divergent trajectories of stress, coping, and collective engagement in everyday life under accelerated transformation (Vestergren et al., 2022; Wannewitz et al., 2023). Habitus–field mismatch does not translate uniformly into suffering; it can stabilise into at least two response regimes. An anomic regime is associated with shame, withdrawal, and closure of the space of possibles (Doblytė, 2022; Weatherford et al., 2024). A radicalising regime is associated with the collectivisation of grievance, moral indignation, and organised participation, without implying violent extremism (Vestergren et al., 2022). This typology brings into a shared analytic vocabulary bodies of work that are frequently treated in isolation, including research on psychological distress and deterioration in well-being, and scholarship on mutual aid, collective action, and resilience (Birk et al., 2023; Voorheis et al., 2023).

The paper’s contribution is to introduce a regime-based model of social hysteresis. Regime-based, path-dependent formulations are increasingly used to capture multistability and asymmetric switching in social dynamics (Doniec et al., 2022; Sznajd-Weron et al., 2024). We apply this logic to habitus–field mismatch and suicidal vulnerability, specifying switching between response regimes. The core thesis is that comparable structural strain can yield divergent risk states (Gou et al., 2025; Sznajd-Weron et al., 2024) because mismatch may stabilise either into an anomic processing regime that directs harm inwards, or into a radicalising regime that reframes distress as grievance and anchors agency in organised affiliation, producing non-linear and partially irreversible trajectories (see Cero et al., 2024; Defayette et al., 2024; Weatherford et al., 2024).

Second, this work applies the framework to the limit case of suicide and specifies a hysteretic aetiology of voluntary death. Suicidal risk *R*_t_ is treated as a function of contemporaneous mismatch *S*_t_ and the accumulated history of prior mismatches $H_{t-1,\ldots ,t-n}$ (see Doniec et al., 2022; Gou et al., 2025). Prolonged residence in the anomic regime, characterised by centripetal directionality of harm, increases the probability of suicidal outcomes. Entry into a radicalising regime can attenuate risk by reorienting harm towards the field. The article’s objective is to operationalise the proposed model for suicidal behaviour and daily well-being, derive empirically tractable expectations, and inform interventions capable of acting on contemporaneous mismatch and on the institutional conditions that structure trajectories and regime transitions.

### 1.1. Habitus and Well-Being

Any analysis of contemporary well-being needs to begin from the historically constituted character of the agent–environment relation (Birk et al., 2023; Voorheis et al., 2023). Mental-health crises that currently dominate clinical and media attention should be situated within shifts in social temporality and in the fit between embodied dispositions and objective structures. Bourdieu’s practice theory provides an analytic vocabulary for this task, particularly through the concept of hysteresis.

In Bourdieu’s formulation, habitus can be defined as a system of durable and transposable dispositions that condense agents’ social histories into schemes of perception, evaluation, and action (Bourdieu, 1990; Leeds, 2024). These schemes enable pre-reflexive practical coordination with the environment: they organise anticipations, judgements of likelihood, and assessments of salience without requiring explicit deliberation. Bourdieu refers to “ontological complicity” when subjective structures and objective structures remain sufficiently congruent for the social world to be experienced as stable and predictable. Such congruence presupposes continuity between past conditions of socialisation and present conditions of action.

When that continuity is disrupted by economic crises, accelerated technological transformation, or intense biographical displacement, hysteresis emerges. As Bourdieu argues (Bourdieu, 1977, 1999; Enderle, 2024), habitus exhibits constitutive inertia: dispositions tend to persist after the field has altered its structuring principles. Agents continue to apply schemes calibrated to earlier field configurations, producing systematic misfits between practical expectations and effective opportunities. This generates losses of practical efficacy and weakens correspondence between embodied competencies and environmental demands.

Recent work has clarified the empirical relevance of this mechanism. Strand and Lizardo (2017), in their reconstruction of the hysteresis effect, argue that habitus–field mismatch disrupts practical coordination in everyday life. Doxa, understood as tacit assumptions about how the world works, becomes unstable, pushing agents towards intensified reflexivity to orient action (see also Doblytė, 2022). Convergent work in formal modelling treats hysteresis as system memory and shows that recovery can be delayed when adaptation is history-dependent (Sznajd-Weron et al., 2024).

This paper takes these developments further by introducing a regime-based taxonomy of social hysteresis (Doniec et al., 2022). It distinguishes an anomic regime and a radicalising regime as alternative stabilisations of mismatch and uses this taxonomy to derive implications for daily well-being, stress dynamics, and suicidal vulnerability.

### 1.2. The Somatic Dimension of Hysteresis

The somatic dimension of hysteresis warrants explicit emphasis. Within a daily well-being perspective, habitus–field mismatch can be treated as an erosive force acting on ontological security (Gou et al., 2025; Morley et al., 2025). Bodily *hexis*, including posture, sleep organisation, breathing patterns, and physiological stress responsivity, provides a substrate on which tensions generated by an environment experienced as illegible are inscribed (Christensen et al., 2022).

Research on chronic stress suggests that prolonged exposure to persistent strain can contribute to elevated allostatic load (McEwen & Stellar, 1993). Recent large-scale and review evidence supports the plausibility of “scarring” pathways that endure beyond short-term changes in objective conditions. In population-level data, perceived stress is associated with higher odds of elevated allostatic load (Morley et al., 2025), and prospective evidence links higher allostatic load to elevated subsequent risks of depression, anxiety, and suicide (Gou et al., 2025). Sleep is one plausible channel through which these processes are embodied. A recent systematic review and meta-analysis links sleep disturbance and sleep duration to allostatic load, reinforcing the relevance of sleep disruption as part of the somatic imprint of prolonged strain (Christensen et al., 2022).

Self-conscious emotions are also relevant because the anomic regime foregrounds shame and inwardly directed harm. Recent meta-analytic work has begun to clarify neural signatures of shame, embarrassment, and guilt, indicating partially distinct activation patterns and underscoring the need to treat these emotions as specific processes rather than generic negative affect (Piretti et al., 2023). Methodological and ecological constraints in studying self-conscious emotions further motivate designs closer to daily life and sensitive to within-person dynamics (Singh & Bhushan, 2025). Taken together, these strands support a behavioural account in which mismatch can become embodied through stress physiology, sleep disruption, and self-conscious affect, and can thereby persist even when external conditions partially improve. In the terms of the present framework, such embodiment helps explain why prolonged residence on the anomic branch can become self-reinforcing, sustaining elevated vulnerability despite partial reductions in contemporaneous mismatch.

## 2. Materials and Methods

This paper follows a theory-building, concept-driven design (see Birk et al., 2023) aimed at (i) clarifying social hysteresis as a mechanism of habitus–field mismatch, and (ii) specifying how this mechanism can generate divergent, path-dependent trajectories in daily well-being and suicidal distress. The contribution is analytical and integrative rather than empirical: no new primary data are collected. The outputs are (a) a regime-based typology (anomic vs. radicalising), (b) a schematic dynamical representation of path dependence (hysteresis), and (c) a set of operational propositions intended to guide future empirical testing and intervention design.

### 2.1. Scope and Source Strategy

The framework is intended for behavioural and social-science researchers, and for applied analysts working at the interface of mental health, community interventions, and social policy. It is most appropriate when the research or applied question involves rapid social change or recurrent structural shocks that plausibly alter the “rules of the game” in relevant fields (Birk et al., 2023); evidence or expectation of habitus–field mismatch (persistent practical misfit between embodied expectations/competencies and current environmental demands); and divergent trajectories under similar exposure, including persistence of distress after objective conditions partially improve. The framework is not intended as an individual-level clinical prediction tool; its role is to generate mechanistic hypotheses, measurement strategies, and intervention targets linking structural strain, response orientations, and distress trajectories.

To build the explanatory mechanism, we used an iterative, purposive source strategy (Voorheis et al., 2023) combining (a) foundational theory on practice, habitus–field mismatch, and anomie/social integration; (b) scholarship on accelerated social change and its psychosocial consequences; and (c) behavioural and clinical research on stress, coping, routine disruption, and suicidal distress. Sources were identified through topic-focused searches and backward/forward citation chaining. Inclusion was based on conceptual relevance to the mechanism under study and on whether a source contributed definitional clarification of a construct, a plausible causal link in the proposed pathway, or an empirical regularity that the framework must accommodate. This strategy is appropriate for mechanism-oriented theory-building (Birk et al., 2023), where the goal is explanatory integration and generation of testable propositions rather than exhaustive coverage.

### 2.2. Model Development Procedure

The framework is developed in three analytic steps that can be replicated by other researchers working on different settings or populations.

Step 1: Mechanism specification (mismatch as a temporally extended condition). We specify habitus–field mismatch as a sustained condition in which embodied expectations (Gou et al., 2025; Sznajd-Weron et al., 2024) and practical competencies lag behind rapidly changing field demands. Social hysteresis is used as the organising principle to capture known features of such dynamics (De Arruda et al., 2023; Doniec et al., 2022) persistence (delayed adjustment), memory (dependence on exposure history), and threshold-like transitions (non-linear switching).

Step 2: Regime identification (response orientations under mismatch). We derive two ideal-typical response regimes (Birk et al., 2023; Zerubavel, 2021) as alternative stabilisations of mismatch, defined by (a) how distress is interpreted and (b) the predominant directionality of blame/harm. *Anomic regime:* distress is privatised; shame and self-blame predominate; withdrawal and isolation increase; harm tends to be directed inward.

*Radicalising regime*: distress is reframed as grievance; moral indignation and external attribution increase; agency is expressed through organised participation and relational anchoring (without implying violent extremism).

These regimes are treated as analytical ideal types, not fixed psychological profiles. They represent attractor-like patterns that can organise experience and behaviour under structural strain, and they may coexist or alternate within individuals over time.

Step 3: Outcome mapping (from regimes to well-being and suicidal distress).

We link the regimes to daily well-being and suicidal distress (Gou et al., 2025; Weatherford et al., 2024) by proposing that sustained mismatch combined with prolonged residence in an anomic regime increases vulnerability to self-destructive trajectories, whereas radicalising processing can (under specified conditions) buffer suicidal risk by providing meaning, social anchoring, and interpretive resources—without assuming that distress disappears.

### 2.3. Model Use, Figures, and Derived Propositions

To make the framework usable, we specify a minimal “theory-to-measurement” workflow (see Birk et al., 2023; Voorheis et al., 2023) that can be implemented in empirical and applied settings. In any given study context, users can: (1) operationalise mismatch dynamics $\left(S_{t}\right)\;$by specifying the relevant field change/shock, time window, and experienced/sustained misfit; (2) operationalise regime indicators $\left(R_{t}\right)\;$by measuring interpretive framing, social anchoring/organised affiliation, and directionality of blame/harm; (3) capture exposure history or “scarring” $\left(H_{t-1,\ldots ,t-n}\right)\;$by recording prior mismatch episodes and regime residence (duration and sequencing), ideally using longitudinal or experience-sampling designs; and (4) test hysteresis-pattern predictions (multistability under comparable $S_{t}$, asymmetric recovery, and threshold-like switching). Regime indicators should be operationalised independently of suicidal outcomes to avoid circularity.

Figures are conceptual diagrams (own elaboration) designed to formalise assumptions and clarify qualitative predictions. They make explicit: (i) multistability (different stabilised regimes under comparable mismatch), (ii) switching thresholds between regimes, and (iii) asymmetry in recoverability (returning to prior well-being states may require stronger restorative conditions than those that preceded deterioration). The figures are not fitted to data.

From the mechanism, typology, and dynamical representation, we derive propositions in a “theory-to-measurement” manner. Each proposition specifies: (a) the structural condition (mismatch dynamics); (b) the regime indicator(s) (framing, anchoring, directionality); and (c) the expected pattern in daily well-being and suicide-risk markers (persistence, threshold shifts, hysteresis effects). These propositions are intended to guide future research designs (longitudinal panels, experience sampling, multilevel modelling linking macro-shocks to micro-trajectories, and comparative analyses of interpretive/support infrastructures).

## 3. Results

A hysteretic aetiology of suicide can be stated as a set of analytic outputs that make the framework operational. Comparable episodes of habitus–field mismatch do not translate into uniform trajectories of daily well-being or suicidal distress. Instead, mismatch can stabilise into distinct response regimes, and suicide risk depends on both contemporaneous strain and accumulated biographical history. The model combines a regime bifurcation, a path-dependent risk specification, and a dynamical representation of multistability and asymmetric switching, followed by brief illustrative applications in concrete contexts.

### Model Outputs and Applications

Under comparable episodes of habitus–field mismatch, trajectories of daily well-being and suicidal distress can diverge along two ideal-typical response regimes. In an anomic regime, distress is privatised and coded as personal defect, increasing shame, withdrawal, and inwardly directed harm. In a radicalising regime, distress is reframed as grievance and agency is supported through organised ties and outward-directed attribution, without implying violent extremism. The key result is that mismatch does not map linearly onto distress. It can stabilise into different orientations that become relatively persistent once established.

Suicide risk at a given moment, denoted $R_{t}$, is treated as an emergent property of the hysteretic system and is not reducible to contemporaneous mismatch $S_{t}$. The model specifies:

$$R_{t}=f\left(S_{t},H_{t-1,\ldots ,t-n}\right),$$

where $S_{t}$ captures the perceived intensity and duration of mismatch in the current period, and $H_{t-1,\ldots ,t-n}$ captures accumulated exposure history, including the duration and sequencing of prior mismatches and prior residence in the anomic versus radicalising regime. This formulation implies that two individuals with comparable $S_{t}$ can display different $R_{t}$ because their histories H place them on different branches of the system.

**Figure 1:**
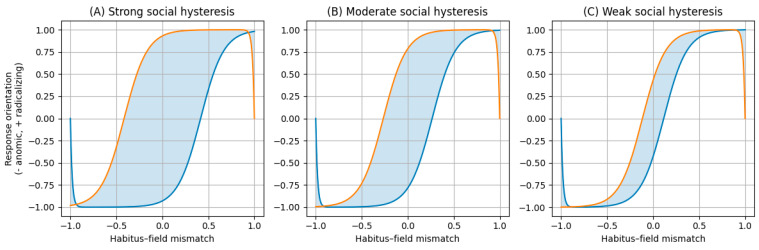
Social hysteresis regimes. Note: (**A**) Strong social hysteresis: wide loop indicating a large region in which the same level of habitus–field mismatch can be associated with either an anomic or a radicalizing response orientation. (**B**) Moderate social hysteresis: intermediate loop width, where transitions between regimes require substantial but still attainable changes in mismatch and support structures. (**C**) Weak social hysteresis: narrow loop, representing contexts of high plasticity in which responses are more easily reversible. In all panels, the horizontal axis represents habitus–field mismatch and the vertical axis represents response orientation (from anomic to radicalizing). The shaded area denotes the hysteresis region, that is, the range in which the outcome depends on the historical trajectory rather than on the current level of mismatch alone. Blue line: lower (anomic) branch. Orange line: upper (radicalizing) branch. Shaded area: hysteresis region (bistability), where the same mismatch value can correspond to either branch depending on the system’s history. Source: own elaboration.

Figure 1 represents the two regimes in a mismatch–response plane. Loop width represents system plasticity and ease of switching. A wide loop indicates a larger region of multistability in which the same level of mismatch can correspond to either regime depending on the trajectory. A narrow loop indicates higher reversibility and easier switching between orientations.

**Figure 2:**
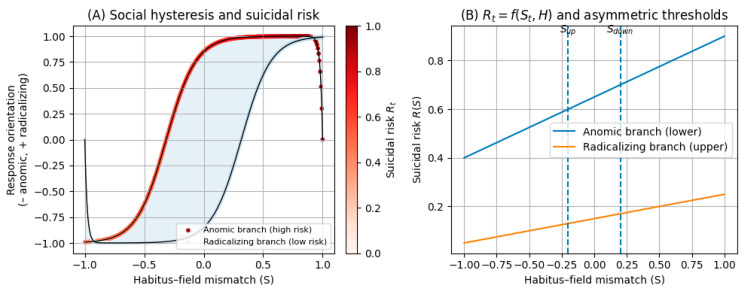
Hysteretic model of suicidal risk. Note: (**A**) Social hysteresis loop in the plane defined by habitus–field mismatch (S) and response orientation (from anomic to radicalizing). The lower branch represents the anomic regime, associated with high levels of suicidal risk $R_{t\;}$ (encoded by the colour scale), whereas the upper branch represents the radicalizing regime, associated with low $R_{t}$. The shaded area indicates the hysteresis region, in which the same level of mismatch can correspond to either anomic or radicalizing states depending on the historical trajectory. (**B**) Suicidal risk $R\left(S\right)$ for each branch as a function of mismatch S. The blue line depicts $R^{\left(\mathrm{anomic}\right)}\left(S\right)\;$ on the lower branch and the orange line $R^{\left(\mathrm{radicalizing}\right)}\left(S\right)$ on the upper branch. Vertical dashed lines mark the asymmetric thresholds $S_{\mathrm{down}}\;$ and $S_{\mathrm{up}}$: beyond $S_{\mathrm{down}}\;$ the system falls from the upper to the lower branch, whereas returning to the protective branch requires reducing mismatch below $S_{\mathrm{up}}$ and/or interventions that modify the hysteretic history H of mismatch. (**A**) The loop shows the two stable branches; markers/line styles distinguish the anomic vs radicalizing branch (as indicated in the legend). The colorbar encodes suicide risk $R_{t\;}$(0–1). The shaded area denotes the hysteresis region. (**B**) Blue line: $R^{\left(\mathrm{anomic}\right)}(S)$ (lower branch); orange line: ${R\;}^{\left(\mathrm{radicalizing}\right)}(S)$ (upper branch). Vertical dashed lines indicate the asymmetric thresholds $S_{\mathrm{up}}\;$and $S_{\mathrm{down}.}$ Source: own elaboration.

Figure 2 links branch occupancy to suicide risk and makes the asymmetry of switching thresholds explicit. The model assumes that, after entry into an anomic state, returning to the protective branch typically requires more than a modest reduction in mismatch. It requires stronger reductions in mismatch and/or changes that modify the history term H, such as restored social anchoring, renewed interpretive resources, and reopened future alternatives. In other words, deterioration and recovery are not symmetric processes.

The following brief applications illustrate how the framework is used in empirical or applied settings, using $S_{t}\;$ for current mismatch, H  for accumulated history and regime residence, and $R_{t}\;$ for suicide risk.

First, consider a post-layoff trajectory. A sudden labour-market shock increases $S_{t}$ through disrupted routines, perceived rule instability, and reduced practical efficacy. In one trajectory, repeated setbacks consolidate an anomic regime, shame and withdrawal increase, and H  accumulates as prolonged residence on the lower branch. Even if objective conditions partially improve, $R_{t\;}$ can remain elevated because the individual remains within the hysteresis region and recovery is constrained by accumulated history. Under comparable $S_{t}$, a different trajectory becomes plausible when organised ties are available early enough to support reframing and affiliation, facilitating a shift toward the upper branch and yielding lower $R_{t}\;$ at the same mismatch level.

Second, consider chronic cohort mismatch in early adulthood under high barriers to housing and stable employment. Here $S_{t\;}$ is shared and persistent rather than acute. The model predicts multistability. Some trajectories stabilise in an anomic regime where blocked transitions are coded as personal inadequacy and future projection collapses, increasing withdrawal and suicidal distress. Other trajectories stabilise in a radicalising regime when interpretive frames and organised affiliation are available, providing recognition and social anchoring that reduce the probability of inwardly directed harm even when mismatch remains high. The observable implication is that similar macro exposure can yield different micro-trajectories because H and regime embedding differentiate branch membership.

Third, the model implies threshold-like switching after cumulative shocks. An individual may remain on the upper branch under moderate mismatch until an additional shock pushes mismatch beyond a dropping threshold, precipitating a rapid transition into an anomic state. Returning to the protective branch then typically requires more than returning mismatch to its prior level. It requires stronger restorative change and/or interventions that alter H, such as restored routines, renewed anchoring, and credible future alternatives. This illustrates the asymmetric recoverability encoded by the hysteresis structure.

## 4. Discussion

The proposed model invites a re-specification of several canonical assumptions in sociological theories of suicide. Within Durkheimian accounts, variation in suicide is primarily related to integration and regulation at a given moment (Durkheim, 1897/2002; Zhang et al., 2024). Temporality is present but rarely formalised as a mechanism, so the question of why recovery might be asymmetric, or why comparable contemporaneous strain might correspond to distinct risk states, remains under-specified. The hysteretic approach advanced here treats the relationship between structural change and suicidal vulnerability as non-linear, history-dependent, and potentially partially irreversible. Suicidal distress is therefore approached as a function not only of contemporaneous mismatch at time t, but also of accumulated exposure history and of the affective–relational response regimes that such mismatches can stabilise over time.

Recent sociological work has increasingly emphasised meaning, culture, and meso-level mechanisms in explanations of suicide, moving beyond accounts that rely exclusively on contemporaneous macro correlates. Formalisations of social disorganisation, for example, foreground how disrupted social ecology and weakened normative supports shape vulnerability (Abrutyn, 2023). Complementary perspectives argue that suicidal vulnerability is mediated by culturally structured meanings and by phenomenological experience, and that sociology must therefore locate the structure–culture dialectic at the level of lived trajectories rather than treat it as a purely contextual background (Chandler & Wright, 2024; Doblytė, 2024; Zhang et al., 2024).

From the perspective of practice theory, Bourdieu introduced hysteresis to designate temporal misalignment between habitus and field (Bourdieu, 1999; Enderle, 2024; Leeds, 2024). Yet hysteresis is most often discussed as a problem of practical intelligibility and declassification rather than as a structured pathway to suicidal vulnerability. Work on reflexivity and habitus–field mismatch has clarified response orientations under misfit (Strand & Lizardo, 2017; Doblytė, 2022), but it has not been systematically articulated with behavioural and clinical processes shaping suicidal distress (Chandler & Wright, 2024).

The present paper advances a regime-based extension that integrates these strands while remaining compatible with contemporary modelling work on social hysteresis. Recent agent-based and related formal approaches treat social hysteresis as multistability, collective memory, and asymmetric switching in social systems, rather than as metaphor, and they provide a vocabulary for thinking about persistence and delayed recovery in a principled way (Doniec et al., 2022; Kamińska & Sznajd-Weron, 2025; Sznajd-Weron et al., 2024). Within suicide-relevant practice-theory scholarship, Doblytė’s empirical work connects hysteretic habitus, closure of the “space of possibles,” and suicide vulnerability, supporting the plausibility of hysteresis as a mechanism in this domain (Doblytė, 2022).

The paper’s contribution is to introduce a regime-based model of social hysteresis that links habitus–field mismatch to daily well-being and suicidal vulnerability through path-dependent switching between response regimes. Building on concept-driven sociology and ideal-typical analytical strategies, we specify two ideal-typical regimes through which mismatch can stabilise (Brekhus & Sabetta, 2024; Zerubavel, 2024). Temporality is formalised by treating suicidal vulnerability $R_{t}$ as a function of contemporaneous mismatch $S_{t}$ and accumulated exposure history $H_{t-1,\ldots ,t-n}$. This formalisation has behavioural relevance because it predicts that identical present conditions may correspond to different risk states, that switching is threshold-like and asymmetric, and that recovery may be delayed even after objective conditions improve.

### 4.1. Implications for the Behavioural Sciences and Empirical Expectations (E_1_–E_6_)

The model implies that structural precariousness and suicidal distress cannot be linked without specifying regime embedding and exposure history. Under comparable mismatch $S_{t}$, anomic trajectories should show higher suicidal distress and risk $R_{t}$ than trajectories embedded in radicalising contexts that provide interpretive resources and organised social anchoring. The model also implies that delayed recovery and scarring are expected outcomes when history H shapes present response. Longitudinal evidence indicates that mental health risk during unemployment is shaped by unemployment history beyond contemporaneous status (Junna et al., 2022), that regaining employment security does not necessarily yield immediate improvements in mental health within persons (Jin et al., 2025), and that re-employment reduces mental health risk on average while leaving substantial heterogeneity in recovery trajectories (Sterud et al., 2025). Prospective cohort evidence further links higher allostatic load to subsequent depression, anxiety, and suicide risk, consistent with embodied pathways of persistence beyond short-term contextual changes (Gou et al., 2025).

At the proximal level, the anomic regime foregrounds self-conscious emotions and inwardly directed harm. Recent synthesis links humiliation and shame processes to later self-harm and suicidal ideation (Sadath et al., 2024), and empirical work shows associations between shame and suicidal ideation and self-harm urges (Weatherford et al., 2024). Neuroimaging synthesis supports treating shame-related processes as specific mechanisms rather than generic negative affect (Piretti et al., 2023). Methodological and ecological constraints in studying self-conscious emotions further motivate designs closer to daily life (Singh & Bhushan, 2025), consistent with the growing use of ecological momentary assessment to capture real-time dynamics of self-harm risk in natural environments (Winstone et al., 2024).

The radicalising regime foregrounds social infrastructure as a condition of switching and stabilisation. Recent work argues that social network structure is itself a plausible suicide-prevention target and reviews mechanisms by which network disruption and topology relate to suicide risk (Cero et al., 2024; Defayette et al., 2024). In hysteresis terms, relational and institutional infrastructures can shape multistability and switching probabilities, narrowing the space in which harmful persistence on an anomic branch is likely.

These mechanisms yield the following empirical expectations (E_x_) implied by the schema in Figure 1 and Figure 2:

- E_1_. Non-linearity with asymmetric thresholds. The association between mismatch $S_{t}$ and daily well-being or suicidal distress should exhibit threshold-like changes, and reversals should typically require stronger or more sustained restorative conditions than those that preceded deterioration.
- E_2_. Multistability and path dependence. At comparable $S_{t}$, distinct stabilised states corresponding to anomic and radicalising regimes should be observable with different profiles of daily well-being and $R_{t}$, and occupancy should depend on exposure history H.
- E_3_. Scarring effects. Following high mismatch episodes, later improvements in $S_{t}$ should not imply symmetric recovery, and $R_{t}$ may remain elevated relative to individuals without the same history.
- E_4_. Directionality of suffering as a proximal mechanism. The effect of $S_{t}$ on $R_{t}$ should depend on whether distress is internalised through shame, self-blame, and withdrawal or reframed with agency and social anchoring.
- E_5_. Everyday behavioural differentiation. Under high mismatch, anomic trajectories should be associated with withdrawal, disrupted routines, and ruminative processing, whereas radicalising trajectories should be associated with affiliation and participation together with greater availability of meaning and agency.
- E_6_. Social infrastructure as a loop parameter. Relational and institutional infrastructures should reduce the probability of stabilising in an anomic regime and facilitate transitions out of it, thereby narrowing hysteresis and reducing harmful persistence.

The same logic implies intervention targets that extend beyond individual-level treatment. If suicidal vulnerability depends jointly on contemporaneous mismatch $S_{t}$ and exposure history H, interventions focusing exclusively on individual coping address only part of the system. Strategies consistent with the model combine reductions in structural mismatch with investments in infrastructures that enable recognition, participation, and durable social anchoring, thereby lowering the probability that structural strain is internalised as self-directed harm.

### 4.2. Limitations

Several limitations follow from the scope and design of this paper. The framework is theory-building and does not estimate parameters or effects (Birk et al., 2023; Voorheis et al., 2023) within this manuscript, so its value lies in the clarity of its mechanisms and the tractability of its predictions. The regimes are ideal types, and empirical trajectories may be mixed or intermittent, making careful operationalisation and validation necessary. Key constructs such as mismatch intensity and duration, regime embedding, and scarring may require proxies that vary across contexts, and triangulation across self-report, administrative indicators, and network measures will be important (Cero et al., 2024; Defayette et al., 2024). The model is not intended for individual-level clinical prediction, and its appropriate use is in mechanism-oriented research and in the identification of intervention targets at behavioural, relational, and institutional levels. Generalisation across settings is likely to depend on field-specific rules, cultural scripts, and welfare arrangements (Abrutyn, 2023; Chandler & Wright, 2024; Doblytė, 2024; Zhang et al., 2024). Finally, reciprocal feedback is plausible, as distress may reshape networks and subsequent exposure (Cero et al., 2024; Defayette et al., 2024), and future work should model these endogenous dynamics explicitly.

## 5. Conclusions

This paper develops a temporally explicit model linking habitus–field mismatch to suicidal vulnerability through social hysteresis. The core claim is that suicide risk is history-dependent. It is shaped jointly by contemporaneous mismatch $S_{t}$ and accumulated exposure history H, mediated by affective and relational processing regimes that organise attribution, meaning, and social embeddedness.

The model identifies two ideal-typical stabilisations of mismatch. In an anomic regime, distress is internalised, self-devaluation and withdrawal increase, and harm is directed inward, yielding higher risk. In a radicalising regime, distress is reframed as grievance and agency is sustained through organised affiliation, yielding lower risk at comparable levels of mismatch without implying violent extremism. Represented as hysteresis loops, these regimes imply multistability, asymmetric switching thresholds, and partial irreversibility. As a result, improvements in macro-level conditions may not translate into proportional declines in suicidal distress when biographical history sustains residence on an anomic branch.

The framework also specifies an empirical agenda. Regime membership should be operationalised independently of suicidal outcomes, using indicators of blame and harm directionality, structural reframing of distress, and embeddedness in organised ties and support networks. Testing requires longitudinal leverage to reconstruct H, detect regime persistence, and identify threshold-like transitions, using panel data, intensive longitudinal designs, and biographical reconstruction. Future work should also examine heterogeneity in thresholds and scarring, and how access to relational and institutional infrastructures shapes switching probabilities.

Clinical and prevention implications follow from the same logic. Because risk depends on both $S_{t}$ and H, interventions focused only on individual symptoms are unlikely to be sufficient under intense structural strain. Clinically, the model motivates attention to (i) persistent shame and self-blame, (ii) withdrawal and loss of social anchoring, and (iii) disrupted routines and sleep as potential markers of anomic residence and scarring. At the same time, it highlights the protective role of durable ties and credible future alternatives. In practical terms, prevention is expected to be most effective when it combines reductions in mismatch through material and existential security with investments in relational and institutional infrastructures that support transitions away from anomic regimes and reopen the space of possibles.

## Data Availability

The original contributions presented in this study are included in the article. Further inquiries can be directed to the corresponding author.
